# The Biological Production of Spacetime: A Sketch of the E-series Universe

**DOI:** 10.1007/s10699-023-09908-x

**Published:** 2023-03-27

**Authors:** Naoki Nomura

**Affiliations:** Graduate School of Social Sciences and Humanities, Nagoya City University, Nagoya City, 467 Japan

**Keywords:** E-series universe, Boundary operation, Limit setting/transgressing, Spatial practice, Spacetime code, *Umwelt*, Gregory Bateson

## Abstract

Space and time, which should properly be taken conjointly, are both communicatively produced and created with certain contextual perspectives—they are not independent physical entities. The standpoint of production makes the relationship between space and time comprehensible. They can either be *mental-subjective*, *physical-objective*, or *social-intersubjective*. *Social* and *intersubjective* (or *E-series*) spacetime might shed new light on biological thinking. For general readers, this paper provides a clue regarding an alternative conceptualization of spacetime based on biology.

## Introduction

This paper attempts to provide a reasonable explanation of how biological space and time are bound to one another. It paints a picture of how space and time are connected and coordinated in living domains in the form of ‘spacetime.’ Specifically, the paper’s main goal is to sketch and illustrate the *E-series* spacetime that is unique to the context of interactive biological fields.

The present paper is written in the framework for communication proposed by Gregory Bateson in that it views meaning-making as the key explanatory principle, and it considers interaction, not logic, to be the central universal concern (Bateson, 1972, 1979). Meaning corresponds roughly to information that is qualitative in nature, and the minimum unit of such information consists of not quantifiable *bits* but rather “a difference which makes a difference” (1972, pp. 459–460). This framework does not assume a genuinely independent reality since all living beings, including humans and sea anemones, think in terms of stories, i.e., a series of meanings or positions pertaining to their own circumstances (1979, pp. 14–15). Here, the individual mind (i.e., idea) is not transcendental but rather *immanent* in each body as well as in various pathways and messages outside the body (1972, pp. 467–468, 472–473). What follows is my version of this view.

We have argued that biologically speaking, time can be a semiotic/communicative product instead of an absolute physical entity (Nomura et al., 2018, 2019, 2020; Nomura & Matsuno, 2016). Without the organism’s marking of boundaries (*punctuation* or *limit setting*), time does not occur. Whatever else may be true about time phenomena, it seems obvious that boundaries are always employed. Time is a type of boundary marking.

Consider two types of time that can easily be conceived. One such type is *objective* clock time, and the other is *subjective* individual personal time. There is no scientific way to conclude that clock time exists as a physical reality. Whether we refer to our *subjective* time, which is usually expressed in terms of the past, present and future, or to the globally synchronized *tenseless objective* time of a mechanical clock, both time series are produced by *punctuations* or *limit setting* in the context of an event or a state—but with different modes (i.e., grammars) of delimitation.

The mode of *punctuation* or *limit setting* differs depending on differences in the type of time in question, which we call time ‘series.’ McTaggart (1908, 1927), although he starts with an ontological concern, theorizes about time and divides it into the *A-series* and the *B-series*. Our theory of time borrows his terminology and uses his language to describe the two different series of time without resorting to ontology. That is, the *A-series* corresponds to an individual’s personal practice of time and features such *tenses* as past, present and future, while the *B-series* corresponds to the impersonal, mechanistic practice of *tenseless* sequential time, such as that of a mechanical clock. There is no past, present or future in clock time.

In our communicative framework, McTaggart’s view of time in the *A-* and *B-series* is transformed to the notion of *time codes*—they are no longer terms that denote physical entities but rather the names of communication codes. In addition to the *A-* and *B-series*, we identify a new code in the domain of interaction: the *E-series*, which focuses on the communicative efforts of living organisms (or humans) to adjust timing and move toward mutual synchronization (Nomura et al., 2019, 2020, 2018). We use the term “timing,” which transforms “time” into a verb using the progressive form of “ing,” thereby suggesting a new domain (i.e., *intersubjective* time-*ing*) that cannot be expressed in terms of either *A-* or *B-series* time. A pair of dancers must time each other’s steps according to their perceptions of the partner’s movements. Time is *measured interactively*—i.e., neither by their own individual timing nor by the objective time indicated by their wristwatches.

## Producing Time in the *A-, B- *and *E-series*

Let me summarize how the different series of time set limits and surmount them in each case of the *A-, B-* and *E-series*. Corresponding to the following discussion, Table 1 is presented to the reader.

**Table 1:** Time as a meaning making system

Time	A-series time	B-series time	E-series time
Grammar (Mode of punctuation)	Tense (past-present-future)	Sequence (earlier/later)	Co-adjusting
Code (Clock)	Subjective, internalized individual code	Objective, externalized global code	Intersubjective relational code
Method of timekeeping	By memory and anticipation	By global-synchrony	By local-synchronization
Timekeeper	The first-person agency	The third-person observer	The second-person negotiators

Based on Nomura et al., (2018, 2019, 2020)

The *A-series* represents a *subjective* time code and assumes a single agent that has access to its own memory and anticipation. Our personally felt time based on *tense,* such as the past, present and future, is a good example. Since *tense* is a linguistic property, the *A-series* is applied primarily to humans, although it does not completely exclude organisms that have short, limited memories. When such a flow of time is sensed by a *first-person agency*, time appears in the *A-series*. Time boundaries in this series are set and crossed using the individual’s *tense scale* or the *memory-anticipation scale*. Without surmounting the limits of tense, the individual cannot proceed to the next movement in this time frame. For example, people who experience traumatic incidents often remark, “Time has stopped for me since that day,” indicating their difficulty surmounting the time boundary (Young, 1997), the experience of which is nothing but real for the *first-person agency* in question (See Table 1).

The *B-series* refers to the *objective* time code that is read by an outsider, *the third-person observer*. The *B-series* is a quantitative index. It is characterized as *tenseless*, and its grammar, the mode of *punctuation*, is based on earlier-later (or before-after) relations; accordingly, its movement is Newtonian—always proceeding in one direction. *Global synchrony* is a feature of this series, and mechanical clocks are an example par excellence. When the delimited sequences of events are viewed from the perspective of earlier/later events, time in the *B-series* appears to the observer. The boundaries have already been set and fixed by physicists with rigorous intervals between seconds; accordingly, the boundary marking that occurs in the *B-series* is automatic, crossing the various delimitations indifferently without the participation of any organism. The agency driving *B-series* time may either be that of the physicist (i.e., ‘physicalist’) or an unknown “*it*.” The *B-series* code is useful for human social life, mass production, and modern science; however, it also places us under the control of impersonal, linear time, which is equated with capital. *Momo*, the girl in M. Ende’s novel (1973), fights against the time thieves, who seduce her friends to give up their friendship to save their “time”.^1^

The *E-series* represents the *intersubjective* time code in which organisms coadjust temporal spans in coordinated rhythms and movements. When one individual conjointly determines the timing of the *limit setting* with other individuals via interaction, the effects are reciprocal, and this process requires a different type of time, namely, the *E-series*. This series refers to the interactive time used by *the second-person negotiators* observed by the participants. Migrating birds flying in formation, a large number of sardines swimming together, bacterial *quorum sensing*, and a pair of human dancers all synchronize their steps and actions in the context of this time series. Examples of the *E-series* code exhibit a local synchronization of movement among the players that contrasts with the *objective* time of the controlled global synchrony in the *B-series*. Boundary marking in the *E-series* is interactional or dialogical, and so the determination of the timing for setting and transgressing limits results from negotiated transactions (communication) among the participating organisms.

Due to the interactive operations in boundary marking that occur in the *E-series*, the coordination cannot be exactly concurrent but must be slightly misaligned (i.e., only slightly and momentarily out of tune) (Nomura et al., 2019). This fact may be a creative and nonmechanical aspect of interaction, which permits *E-series* time to have a certain depth of meaning or a degree of semiotic freedom (Hoffmeyer, 1996, pp. 61–66), and it offers the participants room for trial and error. The participants must read the very near future to anticipate, act upon and synchronize it with their partners. The causal relation is thus reversed—it is *retrocausal*; that is, the near future becomes the retroactive cause of present behavior (Matsuno, 2016; Nomura et al., 2019). Unlike the *A-series,* in which the present is located on the *memory-anticipation scale*, boundary marking in the *E-series* consists of mutually predictive movements, i.e., a ‘dance’ of *negotiated anticipation,* from the standpoint of the very near future to ensure its continuous pursuit of the unattainable and limitlessly narrow *now*.

## Space

This paper also views space not as a physical entity but rather as something that is acted upon or carried out on. Space is not a passive receptacle that indifferently takes in objects to the extent permitted by its container capacity. Space—whether mental, physical or otherwise—*does not have an objective existence*. It is continually produced and practiced. Therefore, my concern is not so much the question “What is space?” as “What is *spatial practice*? What are *spatial practices* among humans and other organisms? How do we organisms practice space in our environment?” Philosophical issues such as the question of “what space is” may be quietly bypassed in favor of this constructive stance.

When we consider the term *space*, our conception must involve an agent who has a particular relationship with that space—whether it is one’s workplace, purchased land or a margin left at the bottom of a typed page. I adopt the term *spatial practice*, following Lefebvre (1974), because space calls for an actor to work it out, thereby excluding philosophical ontological contemplation of what space is from the present paper. *Spatial practice*, in which context humans or organisms act directly on their environment in a *dialogical interaction*—a dialogue with their environment—is a biological phenomenon that should be examined for its own sake.

In everyday use, the term “space” includes not only *physical* (material) spaces but also *mental* (conceptual) spaces, the latter pertaining to our lived experiences, ranging from the vicarious thrill of an animated movie to the religious precepts to which we adhere. In everyday speech, therefore, space is an inclusive concept that could also offer a different perspective on human cognitive processes; for instance, an individual’s life history or a nation’s history may be viewed as part of the *mental* (conceptual) space of a single individual or collective of individuals who might correspond to each physical aspect of the space: an individual’s bodily space or a national territory. Furthermore, we must acknowledge the existence of *social* collective space, such as festivals and group activities, which emerge only ad hoc due to social circumstances. Thus, whether the space in question is *mental, physical*, or *social*, it features subject players, the insider participants, who constitute the corresponding active agency.

From the viewpoint of ownership based on the perspective of *spatial practice*, private space is contrasted to public space, both of which assume certain participants who have agency to act upon the space in question—these spaces do not exist without active users. Animals and humans have their own visual spaces based on their physiologies, which differs from species to species. Jakob von Uexküll’s visual presentation of the living room as observed by humans, dogs and flies is an apt illustration of species-dependent spatial grasp (Uexküll & Kriszat, 1957, pp. 360–361). In human perception, objects in the room, such as a chair, a table, plates and glasses, a sofa, the floor, a wall, and a lamp, all possess *functional tones*, whereas for the dog, the space is limited to chairs, a sofa, dishes, and perhaps a light that remain in his *functional circle*. For the fly, however, the *functional circle* is further reduced to include merely the dishes and the light (pp. 360–361). Each species possesses and practices its own living space. Viewed from the opposite direction, i.e., from the environmental perspective, the organisms’ spatial grasp can be viewed in terms of *affordance*, i.e., what the environment offers or qualities of the room that communicate opportunities to select certain things (Gibson, 1979).

Consider *proximity*, which refers to interpersonal or interindividual space based on the “social code” (Hall, 1969). This notion may currently be popular due to the use of the phrase “social distancing” during the coronavirus pandemic. “Social distancing” as a form of space requires participants—the notion is incomplete unless the space is practiced. A number of birds perch on an electric wire, keeping a proper distance from one another, which may be the spatial practice associated with their “social distancing.”

In another example, the living ecology of the smallest geographical unit, the *biotope*, refers to an aggregate of plants and animals that constitute an area that is distinct from other areas (Olenin and Dukrotoy 2006). This area is a habitat or living space for a group of organisms that are organically linked to compose a social circle with an assemblage of certain species of plants and animals. The *biotope* theater is sustained and performed by the combination and collaboration of organisms who are the involved actors. Thus, the ecology of the biotope constitutes an enclosed living environment that always assumes a certain set of players, so that its theoretical foundation overlaps with von Uexküll’s notion of the *umwelt*, the world subjectively experienced by an organism.

All spaces require insider participants or active players to conduct *spatial practice*.

## “Spaces” and “Places”

It is necessary to justify space theory based on *spatial practice* because many people may think that “space is just space existing independently right here.” Accordingly, it is helpful to distinguish *spaces* from *places* to sharpen our focus on this concept. A *place*, according to de Certeau (1984, pp. 117–118), is an order of any kind in accord with which elements are distributed in relationships of coexistence; however, a *space* exists when the vectors of direction, velocities, and time variables are taken into consideration. Thus, while a *place* is *a configuration of undeviating positions* that implies stability, *space* is composed of *intersections of mobile elements*. *Space* is in constant transformation and does not require stability.^2^

Thus, in relation to *place*, *space* is similar to a word when it is spoken. A language features an underlying linguistic system, such as vocabulary and grammar, that is relatively stable over a period of time. In contrast, in conversations, speakers take full advantage of the system by exhibiting spontaneous flexibility in their linguistic exchanges. Alternatively, consider a concert hall as a *place*. The architectural designs are set and proper because they are more or less stable over a long period of time. On the other hand, a concert is different—it is a *space*, not a *place*, because it requires certain players and unique audiences with specific movements. These examples are in line with the concept of *spatial practice* proposed by Lefebvre (1974) such that, as de Certeau (1984) puts it, *space* is a practiced *place*.

Streets that are geometrically designed by urban planners turn into daily *spaces* due to the involvement of participants such as walkers and visitors. Architectural containers merely indicate *places*, but active users produce *spaces* out of these artificially designed environments. Alternatively, the classic novel as a text may be a *place* consisting of words and sentences, but it becomes a literary *space*, a fictional world, when it is read. In this contexts, the concepts of *space* and *place* belong to different logical orders or types (Bateson, 1979)—just as acceleration is a more abstract concept than velocity.

Uexküll also provides us with illustrations of the *space/place* distinction by considering a familiar path (Uexküll & Kriszat, 1957, pp. 362–364). A blind man’s dog must guide him home via a certain path. The route along which the dog leads the blind man may be a familiar path that allows them to avoid obstacles that might cause the man to stumble. The familiar path that the guide dog takes may be their collaboratively produced *space*, which is extracted from numerous cartographical possibilities. Uexküll also observes that a young jackdaw has a familiar path, wheeling and retracing its known course for a return flight to its starting point (1957, pp. 364–365). This situation is essentially the same as the use of airport runways, where an approach from the opposite direction is prohibited for safety reasons. These examples illustrate practiced *spaces*. What *place* is to environment, *space* is to *umwelt.*^3^

## Boundary Operations

Although I have shown the distinction between *space* and *place*, we should not neglect their commonality; that is, both *spaces* and *places* are composed of boundaries and outlines. An owned property as a *place*, for example, has limits and boundaries against someone else’s land. Animal territories also have limits and boundaries beyond which trespassing entails a risk of being attacked by the territory holder. When the property or territory is acted out by the owner or by the animal in question, it can be treated as *space*, and if it is plotted on a sheet of paper (by an architect or a biologist), then it becomes a *place*. In either case, boundaries and limits are required to compose *spaces* and *places*. Furthermore, when an architect or biologist begins to act upon the *place* that he or she has drawn, the *place* once again becomes a *space*. *Spaces* and *places* can go back and forth, but boundaries and limits remain and uphold both.

However, there are also differences between the two: unlike *places, spaces* have *boundary operations*. Boundaries and limits for *places* are set and stable, being composed of “objects that are ultimately reducible to the *being-there* of something dead” (de Certeau, 1984, p. 118) since *place*s are environments given, written texts frozen, and ideas and theories fixed. The boundaries of *places* are static and durable, whereas the boundaries of *spaces* are constantly being rewritten via interaction and negotiation. *Spaces* require a proactive stance of directly *working on* their boundaries via remarking and remaking. Let us call these spatial characteristics *boundary operations*. *Places* have no *boundary operations*. When astronomers observe the cosmos, they *work on* the space, and through that work, according to this view, the “dead” *place* becomes a *space*.

Various *boundary operations*: As indicated, *space* is multifaceted; thus, physically measurable *space* is not everything but rather is only one of various *spaces*. To be precise, there are various *spatial practices*, of which the physical/geometric approach to “correct” measurement is only one. Thus, we should inquire into the common characteristics of practicing space across different domains, such as *mental* (*subjective*), *physical* (*objective*), and *social* (*intersubjective*) *spaces*.

*Space*, unlike air or void, always possesses certain editing outlines and contours that must be recognized: the *space* provided by architecture, one’s inner cognitive *space*, animal territories, cyberspace or a simple circle drawn in a plane are all bounded by physical, electrical or imaginary lines that are created either consciously or unconsciously. Even the universe or the cosmos may be a product of physicists’ attempts to measure its size and limitations or may be simply a figment of ancient imagination. In this context, it is relevant to ask whether things ***have*** outlines whether or not we draw them or whether we ***give*** things outlines when we draw them (Bateson, 1972, pp. 27–32).

Additionally, the outer boundary is not everything. Despite the fact that the field emerges through some actions of *spatial practice* taken to preserve the contour and outline of the *space*, a *boundary operation* also punctuates living events. That is, the participants must continue acting, reacting and interacting *within* the boundary, *along* the boundary and *across* the boundary to constitute an event. A single cell, whether unicellular or multicellular, has cytoplasm with metabolic pathways and genetic information transport. The membrane embracing the single cell functions as a selectively permeable barrier that allows some molecules to pass through while blocking others, thus separating the inner cytoplasm from the outer environment. The cell must not cease its internal metabolic activity—the operation of the citric acid cycle, for example—and must establish relationships with other cells by coordinating with them. This approach may be the cell’s way of maintaining its own bodily space in that the living space is maintained by interacting with the environment. These spatial activities have boundary markings and makings in common.

To conclude this section, let me address the *boundary operation* of bacteria. When the population density reaches a certain threshold of concentration, bacteria tend to execute their behaviors collectively, synchronizing the action of all members of the group. This phenomenon, which is known as *quorum sensing*, is how bacteria identify their context, i.e., the group-wide detection of information about the cell density and species composition of the vicinal community. Signaling molecules called autoinducers are secreted and defused outside the cell in large numbers at high levels of cell density, and some of them reenter the bacteria to trigger changes in gene expression, thus making collective action possible (Bassler & Losick, 2006; Rutherford & Bassler, 2012). The *spatial practice* of bacterial *quorum sensing* accomplishes tasks that no single cell can carry out on its own. *Quorum sensing* is a collective *boundary operation*, a spatial operation intended to recognize, differentiate, and draw a boundary: a borderline between the previous state, at which the density for joint action has not yet been reached, and the present state, at which the stage for collective action has been reached.

## Mechanism: How Boundary Operations Create Space

We now come to the question of how organisms create *spaces* out of *places*. As seen in the previous section, the creation of *spaces* out of *places* is a result of the subject players *working on* boundaries and limits, that is, *boundary operations*. What, then, is the essential action of a *boundary operation*? In his pursuit of a new algebra, the mathematician Spencer-Brown said that a *universe* comes into being when a space is severed or taken apart (1972). In our terminology, this sentence may be adjusted as follows: a *space* comes into being when a *place* is severed or taken apart. This practice, i.e., making a distinction, creates a space.

To rephrase this claim even further, marking boundaries on *places* expresse*s* and articulate*s spaces*. While *places* refer to something *being-there* and are thus fixed and ordered, *spaces* are a type of *expression* articulated through meaningful delimitations. The subject players who find the delimitation to be meaningful are, of course, humans and organisms. *Biotopes* and the *umwelt* as *spaces* may not be retained unless they have meaningful boundaries for the participants. Eggshells as physical boundaries may be retained until the eggs hatch, i.e., when embryos observe environmental cues that the cost/benefit ratio is more favorable outside the capsule than inside it (Endo et al., 2019; Warkentin, 2011). Hatching is the embryo’s *boundary operation* or *spatial practice*, which is also meaning-based.

In other words, the actions of *boundary operation* are intended to set the limit of the spatial boundary and then to transgress it (de Certeau, 1984, p. 123). This limit is not a fixed limit; rather, it is a sign. That is, an organism sets a bound and surmounts (crosses) it. The process is repeated so that the organism approaches the next limit to go beyond it as well. Consider a stop sign on the road. The sign tells you to stop your car, but only for a moment, not for a day—despite the fact that the sign literally tells you to “stop.” Passing the stop sign is a *boundary operation* on the part of the driver, thus creating *spaces*. We all notice that the message of the stop sign is ambivalent because it is virtually saying “Go!”.

Let us consider Uexküll’s famous explanation of the behavior of a tick drawing blood from a mammal (1957, pp. 320–326). Sensing the mammal’s odor of butyric acid, the blind female tick in the bush drops upon the animal from the tip of a twig or is brushed off by it. After successfully landing on the animal’s skin, she moves around to find a hairless spot to start gorging herself with the mammal’s blood. For the female tick, the butyric acid, the tactile shock of the landing, and the sensation of heat on the skin are each bearers of perceptual meaning (i.e., *receptor cues*). These three signs appear in succession. The odor of butyric acid as a *receptor cue* is then “extinguished,” in the words of Uexküll, by the next operational meaning (i.e., *effector cues*) associated with jumping on the mammal’s skin. Unless this cue is “extinguished,” the tick ultimately stays in the bush, sensing the odor. The tactile sensation of the landing as a *receptor cue* is then “extinguished” by the operation of finding a suitable hairless spot, and the perceptual meaning of heat on the spot is further “extinguished” by the operation of gorging on the warm liquid. Here, the “extinguishing” of signs corresponds to ignoring the literal meaning of a stop sign.

How does the stop sign example relate to the tick’s perceptual meaning that has been “extinguished”? Notice that the perceptual meaning of the stop sign is also “extinguished” when the driver moves ahead by passing the sign. The sign being “extinguished,” as Uexküll phrases it, should correspond to *limit setting and transgressing*, as de Certeau puts it (1984). Here, it may suffice to state that the limit (boundary) as a sign has a double message: you must stop but must not stop, or you must observe the *receptor cue* but must also “extinguish” it, which is the message of the *effector cue*. Thus, the *boundary operation* plays a double game—it does the opposite of what the boundary says (de Certeau, 1984), as in the cases of ticks or car drivers.

In communications theory, this mechanism of *boundary operation* is explained using terms such as *report* and *command*. That is, the limit or boundary functions as a *report* about the space, i.e., stopping; however, it simultaneously *commands* the transgression to venture past it to move on. *Report* and *command* are the two communicative functions that are basically embedded in a single message (Ruesch & Bateson, 1951, pp. 179–181; Nomura et al., 2020). For example, a cautionary expression such as “Watch out!” both represents linguistic meaning (as *report*) and orders (as *command*) an individual to act in the space in an avoidant manner. A bird’s song conveys its beautiful voice (*report*) but is also an expression of its appeal (*command*) to the female (Cody & Brown, 1969; Luther, 2008).

The reader might have noticed that Uexküll’s *receptor cue* and *effector cue*, de Certeau’s *limit setting* and *transgressing*, and Bateson’s *report* and *command* are all parallel to one another—these three prominent thinkers gave considerable attention to the same point, that is, dynamics regarding *boundary operation*. The stop sign for Uexküll offers the driver the perceptual meaning of stopping the car (*receptor cue*) but also conveys the operational meaning (*effector cue*) of “extinguishing” the sign with the aim of administrating the space. For de Certeau, the stop sign sets a *limit* to movement in the space but also orders the driver to *transgress* that limit by doing the opposite of what the boundary says, which he called the double game. For Bateson, the stop sign *reports* to the driver one level of spatial meaning that directs the driver to follow the sign but also *commands* the subsequent action to act against the sign to complete the *boundary operation*, which could potentially become paradoxical in relation to the previous message, causing the situation to become a *double bind* (1972, pp. 201–227).

In summary, practiced space is not static. Instead, it is in constant flux and lacks inherent stability; otherwise, maintenance cannot be achieved, whether for animal territories or cyberspace, which must be renewed and edited as long as the subjects are involved. Exchange of information via feedback serves as the basis of the transactions that take place both among player-subjects and between those subjects and the environment; it is *dialogical interaction*.

As the *universe* in terms of the cosmos is bounded whether it is constant or expanding, the *umwelt* that is the organism’s subjective environment corresponds to each organism’s model of the world. Organisms engender and reshape their own environment, the *umwelt*, which is their *functional circle* that is maintained through *boundary operations.* Such agential space or practiced space has meanings and relevance for organisms as participants. Furthermore, when two or more different species interact, they create the mutual perceptual space of the semiosphere (Hoffmeyer, 1996), which is the result of mutual, collaborative *boundary operations*.

## Spacetime as Boundary Operation

“Space” often indicates “time,” such as when we say, for example, “in the space of a moment.” Time and space are intertwined in our language. It is not easy to grasp how these concepts relate to each other because *spatial practice* necessarily entails temporal duration, without which no recognition of space is possible, such as in the examples of an astronomer gazing at stars or a dragonfly chasing its prey. We organisms live in space/time together, not separately.

However, the difficulty may reside in our split—we can grasp spacetime in action and performance, such as in dancing or avoiding an approaching vehicle, but we are intellectually trained to view time and space as separate. Nevertheless, the idea of *boundary operation* connects space with the time series—whether the *A-, B-,* or *E-series*. Both space and time are produced and constructed—they are expressions (i.e., time codes and space codes) of different orders using *limit setting* and *transgressing*.

If *spatial practice* is carried out by *boundary operation*, it may be natural to assume that a *spatial practice* requires the right type of time that measures up to the practice in question because the space’s boundary marking requires a temporal duration. If each time practice is performed via a *boundary operation*, it may also be inevitable that each time practice entails the right type of space because the time’s boundary marking requires such a space for its operation, i.e., it requires qualitative event experience for mental space (the *A-series*), quantity for physical space (in the *B-series*), and interaction for social space (the *E-series*).

Thus, each spatial practice, whether *mental*, *physical*, or *social*, seems to require a certain type of time, i.e., the *A-, B-,* or *E-series*, that has an affinity for each corresponding space, thereby producing something like *A-, B-,* or *E-series* space. This relationship should remain the same even when its direction is reversed. The following passages examine this claim in detail.

*A-series* spacetime code: Marking and crossing boundaries on one’s *memory-anticipation scale* in *A-series* time suggests the production of a type of mental-psychological space that may be called *A-series* space. Recall a time in your childhood when you played with your favorite toy. Such temporal recollection is story-based. It is not the *tense scale* itself that produces space—it is rather the production of events, more precisely, the story, the working out of one’s *tense scale*, that constitutes the *mental* space and in turn makes it “visible” to the subject him- or herself (Ricoeur, 1984).

The subjectively constructed *A-series space* may comprise an organism’s individual *spatial practice*, which enables it to create its own perspectives on its environment. This environment may be the *umwelt* as seen by the individual organism in question in the *first person*. Securing territory, chasing prey, or reading a novel can be examples of this situation. The actor’s cognitive or perceptual *universe* is distinct from those of others.^4^

In this subjective space, organisms should produce time in the *A-series* since the clock used to measure time in the organism’s idiosyncratic *universe* is *internalized* within its own bodily space. If an organism starts beating rhythms with its own internal pace in the *A-series* time code, the code is naturally interpreted in such a manner as to ensure that the spatial code aligns with it.

The narrative space of human life (i.e., personal narrative) falls into this category and has its own unique scope, which is described by the individual’s measurement of temporality (Lewis, 1961). In a narrated history, some past events may be highlighted while others are nonchalantly skipped—the narrative appears to be uneven from the perspective of an objective historian, but the clock that measures an individual’s subjective time constitutes a unique and idiosyncratic *mental* space, such as each person’s memory of his or her old toy.

*B-series* spacetime code: The *objective* time coding of the *B-series* may assume geometric space (i.e., Euclidean space). Humans codify the observation of natural physical oscillators such as the rotation of the earth on its axis or the resonance frequencies of atoms to compose this time series. Thus, the *B-series* time code and space code may be human-specific due to our codification. This code comprises both time practice based on global synchrony and *spatial practice* measuring physical dimensions and proportions. Land surveying, counting speed and distance, or designing architecture are some examples of this situation.

Humans produce the *B-series* time code for *objective* (*physical*) space since the clock used to measure time in the physical/astronomical universe should be *externalized* outside the body. This globally synchronized mechanical clock shows the measurement time scale in *objective* space.

The person who practices *objective* space with *B-series* time is situated outside the associated phenomena. A spaceship must share time with the space center on the ground to conduct its operations in the outer atmosphere. The use of a mechanical clock in this space does not permit the individual sense of *tense*—instead, what is required is universal synchrony, a mutual timeframe that permits the astronaut to coordinate his or her actions with ground personnel. *B-series* time is an a posteriori acquisition of humans that is not shared by other species on earth.

The *E-series* spacetime code is imperative to ensure that the prediction of the very near future does not lag behind the right timing for mutual synchronization among partners in an interaction, without which accurate timekeeping and time assessment may be difficult (Condon, 1970). Note, however, that in reality, such predictive movements require spatial operations. Pedestrians on a busy street avoid colliding with each other by predicting and calculating timing and distance. A flock of birds and a school of fish exhibit the same dynamics when flying or swimming together. Thus, the *E-series intersubjective* time necessitates the production of such *social* space, which is neither *physical* nor *mental*.

*E-series* space focuses on the *spatial practice* of organisms that communicate and interact with each other in the context of social activities or for the purpose of creating bonds and relationships (Tinbergen, 1953). Examples of this situation include human conversation and dancing, puppies playing together, a school of fish swimming, and a male bird’s song and the female responses; these timing works all take place in the interactive social space. The essence of this *spatial practice* is the adjustment of timing (i.e., intersubjective *time-ing*) or synchronization among the engaged organisms.

How is time produced in a *social* or *intersubjective* space? *Intersubjective* space, which covers a vast arena of communication, requires a distinct time for organisms to interact with their environments—with other individuals, with their surroundings and with themselves. Unlike stable *B-series* space, *E-series* space is always in flux and uncertain, thus allowing for constant movements, as is the time that is appropriate to this space.

**Fig. 1:**
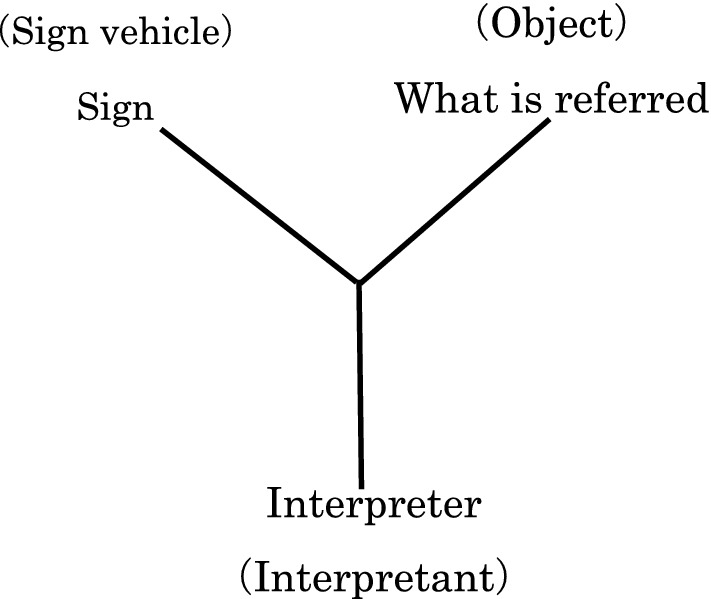
The general sign triad (Hoffmeyer, 1996, p. 19)

The critical point concerning spacetime production pertains to where the organisms, i.e., the agencies, participants, insider-players, or interpreters, are present. This aspect is well illustrated in the *sign triad*, i.e., the logic advanced by Charles Sanders Peirce, according to which the interpreter reads a sign to obtain meaning or to take an action (Hoffmeyer, 1996, pp. 16–24) (See Fig. 1). For example, a doctor (the interpretant) interprets a child breaking out in a rash of red spots (the sign vehicle) to indicate measles (the object). The diagnosis is a cognitive action taken in response to this sign. Alternatively, a car driver (the interpretant) stops at a stop sign (the sign vehicle) and then passes it, having completed its symbolic message (the object).

**Fig. 2:**
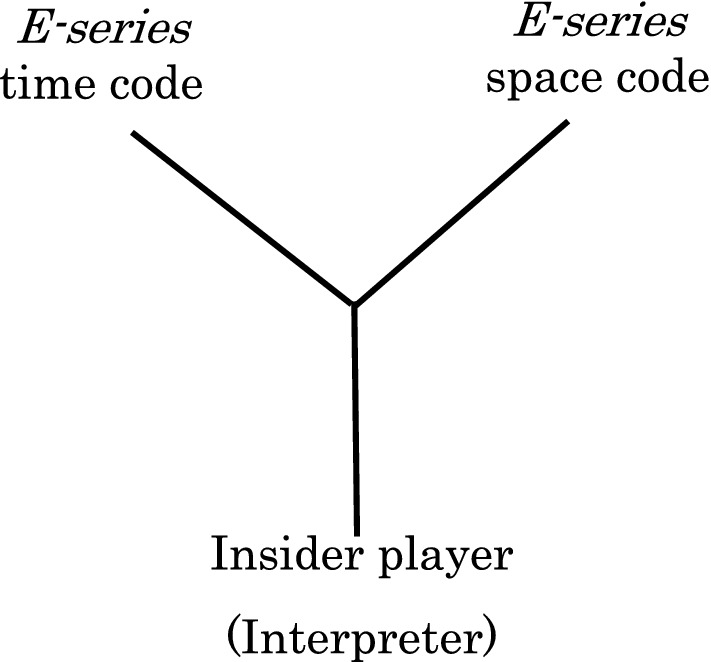
The sign triad applied to the *E-series* time code and space code

In Fig. 2, the *E-series* time code is seen in the sign vehicle on the left, which the interpreter reads and transforms to a space code (on the right) that is in line with the time code, similar to the act of diagnosing an illness. Here, the Peircean logical structure, the *sign triad*, is translated into the language of communication. The sign drives the interpreter to take an action. Thus, the time code as a *receptor cue* is “extinguished” by the interpreter’s acquisition of a space code, which is the operational meaning of the *effector cue*; it can similarly be read as a *limit* being *set* (time code) and *transgressed* by the interpreter to reach a space code or as a message (time code) that *reports* its content and *commands* the interpreter to perform an action, i.e., conduct a spatial practice, such as the driver passing a stop sign.

**Fig. 3:**
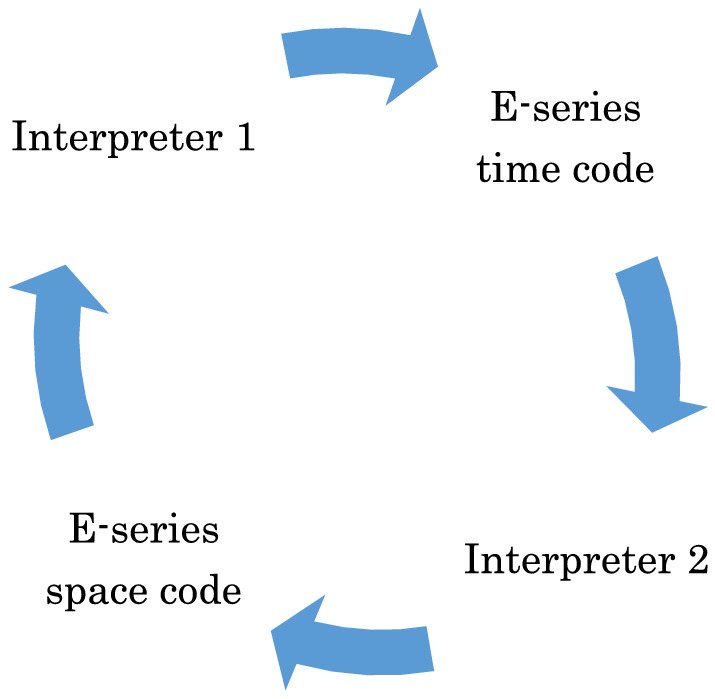
The sign triad converted to the interactive cycle with two interpreters: An image of practicing *E-series* spacetime

Since the interactive *E-series* assumes two (or more) insider-players or interpreters, the two *sign triads* (a *sign triad* for each interpreter) may be combined to form one representation, in which context the relationship is redrawn in a cyclic pattern (see Fig. 3). The diagram includes time elements that allow messages to move around the cycle and enable the cycle to repeat itself. This diagram is my current rendition of practicing the *E-series* spacetime or *universe*.

A case for spacetime operation is illustrated. Long-distance migratory birds exemplify *intersubjective* spacetime behavior. Arctic terns (*Sterna paradisaea*) have the longest annual migration ever recorded in any animal, flying a round trip of more than 70,000 km between Greenland and Antarctica (Egevang et al., 2010). They spend approximately 90 days on their southbound migration to meet the austral summer and approximately 40 days, i.e., less than half the time, on their northbound route to return to their boreal breeding grounds. During their outbound journey, they stay for approximately 3 weeks at their North Atlantic stopover site.

Remarkably, researchers have found that the terns exhibit clear synchrony in the timing of their migration, with all of them reaching the North Atlantic stopover site, departing from the wintering area and crossing the equator within a few days of each other. The flock size of migrating terns was less than 15, but there was no indication that they traveled together in the same flocks (pp. 36–37). Despite flying an enormous distance, migrating terns can synchronize their space-season (-time) orientation, knowing when and where they are supposed to be, interpreting the space code to adjust their timing operations and the time code to adjust their geographical (spatial) location (see Fig. 3).

Thus, spatial practice and time practice go hand in hand, and *boundary operation* may be primordial, serving as the basis for all living practices of spacetime.

## *The Umwelt* and Spacetime

The *umwelt*, the world experienced by a particular organism, has spatial implications (Uexküll & Kriszat, 1957), but it is also time-based since it can persist only in temporal networks. Without attention to temporality (i.e., timework), or an accurate reading of timing, organisms cannot sustain themselves in their routine behavior. Circadian rhythms are steadily maintained by many species, including cyanobacteria, and represent their timework, thus highlighting the importance of their adaptation to celestial cues, such as the sun’s movement (Nakajima et al., 2005). The regular seasonal movement of migratory birds covers a vast area, often in a north–south line, as they fly between their breeding spots and wintering grounds (Egevang et al., 2010). The maintenance of one’s subjective space is therefore made possible by reading and creating each local time. Migrating birds primarily read changes in day length to understand the passage of the seasons and synchronize their behavior, i.e., a flight with other individuals that are navigating to the same destination.

Uexküll made it clear that organisms ***sway*** the time of their own world (p. 326); thus, time and space should be discussed as a single unified phenomenon with a biological foundation. When an organism produces a *space* that has a direct bearing on its survival, the organism must not stop reconstructing and renewing the *space* in question because if it is left unattended, neglect will cause it to decay, which can endanger its survival. In the context of animal territories or cell membranes, the continual renewal of *space* is achieved within a finite span of time; *thus, the living space of organisms inescapably requires a temporal duration to sustain survival.*

The significance of time in the *umwelt* can also be discussed from the perspective of rhythms. Organisms often utilize rhythms to guide their temporality and timing; that is, in the course of their physiological changes, organisms have evolved internal rhythms—the rhythms used to sustain their bodily space through oscillation (*punctuating time*). When the rhythm stops, life as we know it ceases, such as in the case of our hearts. An individual organism must acquire the ability to adjust timing within itself, between itself and others and between itself and the surrounding environment. Generating rhythms and adjusting timing are both acts of *punctuation*—*a boundary operation*—that enable organisms to inform themselves and communicate with others in their spatial environment.

Therefore, the spacetime that Uexküll envisioned for the *umwelt* would not be the mechanical *B-series*. Instead, it would be either the *A-series*, i.e., the flow of space and time that are subjectively sensed by *first-person agency*, or the *E-series*, i.e., the *intersubjective*, interactive relational clocks of a social nature that are locally synchronized within their own environments. A question thus arises: should the *umwelt* be viewed as the organism’s *subjective* spacetime in the *A-series*, the *intersubjective* spacetime in the *E-series*, or both? Let us make a small detour to address this question.

When a distinction occurs in chaos or in the void, such as the new development of a cell membrane or an embryo in the egg or a situation in which an individual simply draws a circle on the ground around him- or herself, a demarcation is created between inside and outside or between self and nonself. This distinction represents “a difference which makes a difference” for an organism. Such boundary marking, whether plasma membrane, eggshell or line on the ground, instantly assumes a distinction between two types of relationships, inside and outside: the relationship with oneself and the relationship with the outside.

Now, if the observer sits shuttered within, the *universe* appears to extend from the inside to the boundary, outside of which a different *universe* prevails. Time within the cell, i.e., within the egg or within the line, must be covered by its own independent temporal pace. This situation may be parallel to Uexküll’s claim that any organism is enclosed within a soap bubble—all organisms remain permanently surrounded by their own soap bubbles, which define their own space, so that there is no space independent of subjects (pp. 28–29). Although these situations may correspond to the *A-series*, such a circumstance may be somewhat unlikely in reality, since the cell, the embryo or the individual within the circle naturally has inescapable relationships with the outside by means of cell-to-cell communication (Neitzel & Rasband, 2021), *environmentally cued hatching* (Warkentin, 2011) or simply talking with someone outside the circle (Sacks et al., 1974). Organisms do not live without context—their surroundings or the environment.^5^

If an observer sits outside as a nonparticipant, the situation appears quite different. The segment of the *universe* becomes a detached entity that can be measured by imposing objective scales, i.e., those of distance and time, on the entity. The attitude by which an outsider-observer relates to physical reality may be definitive and canonical and hence authoritative, in which case the main concern should be whether the measurements, the size of the space and the passage of time, are correct or not based on outside criteria. One example of this situation may be found in children’s development, in which context the developmental stage of a child is evaluated according to a *B-series* spacetime scale on which the child’s age is correlated with intelligence (Piaget, 1953).

However, when organisms create space and boundaries that have a direct bearing on their continued survival, they must continually renew and recreate the boundary—mending and reshaping the parts that require repair—to actively sustain their viability, since their survival would otherwise be endangered. To continue marking and remaking the boundary requires repeated *limit setting and transgressing* and hence involves rhythms and time lapses; accordingly, time and space become intact or equivalent from the standpoint of survival.

On the one hand, the *umwelt* should be a spacetime network that is viewed from the *subjective* point of view of the *first person* within it, a system of language that is used for the internal communication of an organism (Sharov, 2001). The *umwelt* is therefore the ecological niche as the organism itself apprehends it (Hoffmeyer, 1996). On the other hand, modern biology employs the objective term “ecological niche,” i.e., a certain set of conditions in the form of living space, food, temperature, etc., under which the given species lives. This contrast may come from the distinct spacetime orientations taken by these two approaches: Uexküll’s tendency toward personalized *A-series* spacetime versus the depersonalized *B-series* spacetime of modern biology.

However, organisms in the environment also live in *social* and *intersubjective* space with other individuals of the same species, with their prey and predators and with physical objects that are relevant to their survival, all of which constitute the *functional circle* of their *umwelten* based on the processes of communication and interaction. In this context, organisms and relevant physical objects *speak* to each other in this circle, but they do so in different ways, thus forming a network of relationships featuring *reciprocity*, namely, *affordances* (Gibson, 1979). The philosopher Martin Buber (1970) identified the *dialogical* aspect of *reciprocity* with the phrase *I-Thou* (*I-You*), which he contrasted with the *instrumental* communication attitude of *I-It*, which refers to a nonreciprocal relation that exploits and manipulates other individuals and objects. The *E-series universe* and the *B-series universe* correspond to the *intersubjective You-world* and the *objective It-world*, respectively.

It seems evident that the *umwelt* can be viewed as *E-series* spacetime, the *intersubjective universe,* which deploys the *I-Thou (I-You)* perspective of *second-person negotiators*. In this context, a *universe* constructed by the *E-series* code becomes the living spacetime through which the incessant *boundary operations* involved in social interaction ***sway*** time and engender space.^6^

## Conclusion

Spacetime as *universe* has been discussed in terms of a code produced by *boundary operations*. The three parts—space code, time code, and insider-players (interpreters)—compose the spacetime or *universe*. Spacetime without agency may be an empty void or simply a dead domain (De Certeau, 1984). Spacetime can be *mental-subjective, physical-objective,* or *social-intersubjective.* I have suggested how the three types of space code and the three series of time code correspond to one another in biological contexts, thereby constituting the *A-*, *B-*, and *E-series* spacetime or *universe*.

The *E-series universe* is a spacetime code that is produced ad hoc and locally by interaction among participating organisms. Interaction is the key explanatory principle in this context (Bateson, 1972, 1979), and the *intersubjective* spacetime of the *E-series* represents the biological world in which organisms exchange information with others or with their environments to produce their living niches, a local *universe* for each organism. This practice is what creates the *E-series universe*.

## Footnotes

1 The ancient Greeks had two contrasting terms for time: *Chronos*, which conceives of time as measurable based on the quantity of duration, the length of periodicity, or the age of an object, and *Kairos,* which refers to the qualitative character of time and to the special position that an event occupies in a series (Smith 1969). While the former notion resembles the *objective* time code in the *B-series*, the latter is reminiscent of the *subjective* time code in the *A-series*. However, the underlying difference between these two conceptions of time may be that Greek mythology assumes time a priori, that is, the existence of time is taken for granted, whereas time code theory does not make the same assumption.

2 In a classic illustration, the *space/place* contrast is depicted in the following Zen conversation that occurred in the eighth century in China (Tanahashi 2010): A senior monk teaching a junior monk once confronted his pupil: Senior monk: “Do you know how to grasp *space*?”. Junior monk: “Yes, I do.”. Senior monk: “How do you grasp it?”. The junior monk stroked the air with his hand. Senior monk: “You don’t know how to grasp *space*.”. Junior monk: “How do you grasp it, elder bother?”. The senior monk poked his finger into the junior monk’s nostril and yanked his nose. Junior monk, grunting in pain: “You’re killing me! You tried to pull off my nose.”. Senior monk: “You can grasp it now.”. The ancient Zen master already had insight into the distinction between *space* and *place*. The senior monk’s teaching may be abridged to the following: *space* is a progressive field of being alive (and kicking).

3 *Places* transform into *spaces* when the players enter them to engage in social or interactional discourses. An apartment into which one has newly moved becomes one’s living *space*, and the male bear marking a tree trunk indicates his territory, with the land thus becoming his “owned” *space*. In addition, *space* also returns to *place* when, for example, one moves out of the apartment or the male bear leaves his territory for one reason or another. In addition, there may be intermediary stages of ongoing transition between *space* and *place*, such as in the case of acquiring one’s flight path or territory through the process of continuous learning by trial and error.

4 One might note that simple organisms may not have a clear sense of the past, present and future. However, we know that an animal such as a giant anteater (*Myrmecophaga tridactyla*) collecting ants one by one knows what it has just eaten and what it will do in the very near future. A dragonfly chasing a mosquito recognizes where it is flying at the present moment in relation to its prey and anticipates where the prey will be in the next instant (Mischiati et al., 2015). Thus, organisms other than humans can be viewed as possessing a rudimentary sense of *tense*.

5 Uexküll’s spatial concepts, such as *operational*, *tactile*, or *visual* spaces (Uexküll and Kriszat 1957, pp. 14–21), may be considered against the backdrop of *A-series* spacetime since their focus appears to be on the individual.

6 It may be useful to put the notion of *boundary operation* into perspective. *Boundary operation*—boundary marking and making—has been defined in communication-related terms as *punctuation* or as *limit setting and transgressing*, thereby suggesting a theoretical link with semiotic scaffolding. Vigotsky (1986) scheme of the learning process of children influenced the psychologist J. Bruner to coin the term *scaffolding* (Wood et al., 1976); Hoffmeyer (2015) then transferred this notion to the field of biology under the name *semiotic scaffolding* to describe “a *web* of checks and balances,” “what makes *history* matter to an organism,” “*value* as a system property,” and “*matrix* of interactions” (Hoffmeyer 2015, pp. 3–4). These phrases using *web, history, value,* and *matrix* seem to suggest an image of something durable. In relation to *semiotic scaffolding*, the *boundary operation* is similar to the word when it is spoken, which represents the practiced side. This context is similar to my discussion of the contrast between *space* and *place*, which described *space* as a practiced *place*. Each *boundary operation* transaction—the driver cutting across a stop sign, for example—represents a microstep in the process of building *semiotic scaffolding* that organizes the entire traffic network to constitute the whole “*matrix* of interaction.” Furthermore, the term *boundary operation* describes the process by which the *functional circles* of the *umwelt* are formed to constitute “the *web* of checks and balances” and its compatible “*value* as a system property.” While a *boundary operation* is in constant movement and lacks proper stability, *semiotic scaffolding* implies a stable configuration of positions or biological constructs.
